# Enhanced GNSS Navigation Using a Centered Error Entropy Extended Kalman Filter in Non-Gaussian Noise Environments

**DOI:** 10.3390/s26041148

**Published:** 2026-02-10

**Authors:** Yi Chang, Dah-Jing Jwo, Bo-Yang Lee

**Affiliations:** 1 Department of Electrical Engineering, National Taiwan Ocean University, Keelung 202301, Taiwan; ocat3021@gmail.com; 2 Department of Communications, Navigation and Control Engineering, National Taiwan Ocean University, Keelung 202301, Taiwan; 3 Chunghwa Telecom Co., Ltd., Taipei 106001, Taiwan; jacky25089118@cht.com.tw

**Keywords:** GNSS, extended Kalman filter, centered error entropy, minimum error entropy, maximum correntropy criterion, non-Gaussian noise, multipath effects

## Abstract

Global Navigation Satellite Systems (GNSSs) observables, such as those of the Global Positioning System (GPS), are frequently affected by multipath effects that cause unpredictable signal interference at the receiver, posing significant challenges for accurate state estimation in complex environments with non-Gaussian noise or outliers. The traditional extended Kalman filter (EKF), based on the minimum mean square error (MMSE) criterion, assumes Gaussian noise distributions and exhibits degraded performance under non-Gaussian conditions. To overcome this limitation, the minimum error entropy (MEE) criterion was proposed to reduce random uncertainty in estimation error distributions; however, due to its translation invariance property, MEE may inadvertently increase bias when errors contain systematic offsets, leading to poor convergence. In contrast, the maximum correntropy criterion (MCC) concentrates the error probability density function (PDF) around zero, enabling effective entropy adjustment even in the presence of bias and achieving superior error convergence. This paper presents the centered error entropy (CEE) extended Kalman filter (CEE-EKF) that integrates the complementary merits of both MEE and MCC approaches to overcome their individual limitations. Experimental validation in complex nonlinear GPS environments with non-Gaussian noise demonstrates that the CEE-EKF significantly outperforms individual algorithms in noise suppression, particularly exhibiting enhanced robustness and accuracy when handling outliers. These results offer an effective approach to enhancing the reliability of GPS navigation in challenging real-world environments, and the algorithm can be readily extended to other GNSS applications.

## 1. Introduction

Accurate state estimation in Global Navigation Satellite Systems (GNSSs), such as the Global Positioning System (GPS) [1,2], is challenging under conditions involving complex non-Gaussian noise, where measurement outliers and heavy-tailed error distributions can severely degrade positioning accuracy. The Kalman filter (KF) is an optimal linear minimum mean square error (MMSE) estimator for linear dynamic systems under the assumptions of linear state-space models and additive Gaussian noise [3,4]. However, the MMSE criterion implicitly assumes that both process and measurement noise are Gaussian, an assumption that is often invalid in realistic GNSS environments where multipath effects, interference, and signal blockages give rise to non-Gaussian noise characteristics. While the extended Kalman filter (EKF) enables nonlinear state estimation, it remains an approximate MMSE estimator and retains the Gaussian noise assumption. Consequently, its estimation performance may deteriorate in the presence of strongly non-Gaussian noise, motivating the development of more robust nonlinear filtering frameworks.

The use of entropy as a performance criterion in signal processing stems from Rényi’s foundational work on generalized entropy measures [5], which established the theoretical basis for information-theoretic learning (ITL). Extending this framework, Principe [6] introduced kernel-based representations of Rényi entropy, enabling practical estimation of information-theoretic cost functions and their application to nonlinear filtering and machine learning problems. To address the limitations of the Kalman family of filters under non-Gaussian noise conditions, the minimum error entropy extended Kalman filter (MEE-EKF) was proposed [7,8,9,10,11,12,13,14,15,16,17,18,19,20]. Unlike MMSE-based methods that minimize second-order moments, the MEE-EKF minimizes the entropy of the estimation error, thereby capturing higher-order statistical information and improving robustness in non-Gaussian noise environments. Despite these advantages, existing studies have reported that MEE-EKF may suffer from numerical instability or even divergence under certain system dynamics and parameter settings.

Wang [7] employed the minimum entropy criterion to non-Gaussian stochastic system control and demonstrated that entropy-based objectives yield robust performance in the presence of uncertainty. Indiveri [8] introduced a least entropy-like filtering method for range measurements with outliers, demonstrating the effectiveness of entropy measures in suppressing impulsive noise. Chen et al. [9] proposed the kernel minimum error entropy (KMEE) algorithm, extending the MEE criterion to nonlinear estimation using kernel density estimation. Subsequent studies incorporated the MEE principle into Kalman filtering frameworks, including the standard Kalman filter and the unscented Kalman filter (UKF), demonstrating enhanced robustness against non-Gaussian disturbances [10]. KMEE-based approaches were subsequently employed for multipath estimation in navigation systems, demonstrating their practical effectiveness [11]. Luo et al. [12] provided a theoretical link between the Kalman filtering framework and Rényi entropy, reinforcing the information-theoretic interpretation of MEE-based estimators. More recent efforts have focused on improving the numerical stability and robustness of MEE-based Kalman filters [13,14,15,16] and extending them to packet-loss scenarios [17], distributed estimation architectures [18], and power system state estimation [19]. Feng et al. [20] proposed Wasserstein distribution-based MEE filtering methods, further enhancing robustness under model uncertainty and non-Gaussian noise.

As an alternative to entropy minimization-based approaches, the maximum correntropy criterion extended Kalman filter (MCC-EKF) was introduced to enhance robustness against non-Gaussian noise by maximizing the correntropy of the estimation error [21,22,23,24,25,26,27,28,29,30,31]. As a localized similarity measure defined in a reproducing kernel Hilbert space, correntropy emphasizes higher-order statistical moments and effectively suppresses impulsive disturbances. Although MCC-EKF exhibits strong noise rejection capabilities, its performance may degrade in highly dynamic systems due to sensitivity to kernel parameters and reduced adaptability to rapid state variations. In conjunction with MEE-based approaches, the MCC framework has received significant research attention, with notable contributions including Izanloo et al. [21], Liu et al. [22], and Chen et al. [23], followed by subsequent extensions to extended, cubature, and multi-kernel correntropy-based Kalman filters [24,25,26,27,28,29,30,31]. These developments demonstrated improved robustness and estimation accuracy in nonlinear systems subject to heavy-tailed and impulsive noise.

A further advancement in entropy-based filtering is the introduction of centered error entropy (CEE) filtering. The CEE-based filter normalizes the estimation error distribution by removing bias. The centered error entropy extended Kalman filter (CEE-EKF) was developed to combine the advantages of minimum error entropy (MEE) and maximum correntropy criterion (MCC), mitigating the individual limitations of each approach [32,33,34,35,36,37,38,39,40,41,42]. By jointly reducing error uncertainty and enhancing noise suppression, the CEE-EKF achieves superior performance in nonlinear systems affected by non-Gaussian noise.

Cheng et al. [32] and Yang et al. [33,34,35,36] demonstrated that CEE-based filtering approaches are effective for satellite attitude determination, spacecraft navigation, and integrated INS/GNSSs, exhibiting strong robustness under non-Gaussian noise conditions. Extending the CEE framework, fiducial point-based extensions of minimum error entropy (MEE) filtering were introduced to further enhance robustness against outliers. For example, Xie et al. [38] and Mitra et al. [39] incorporated strategically selected fiducial points into MEE filters to improve estimation accuracy in localization applications. Bahrami and Tuncel [40] provided an analysis of fiducial point selection and deployment strategies, identifying practical limitations and offering implementation guidelines. He et al. [41] and Zhao and Tian [42] extended these approaches to generalized and distributed MEE frameworks suitable for large-scale, networked, and high-precision state estimation.

In this paper, the CEE-EKF is applied to the GPS navigation state estimation, and its robustness and noise attenuation capabilities are evaluated under challenging non-Gaussian noise environments. Some highlights of the main contributions of this paper are as follows:

- The centered error entropy (CEE) criterion is employed to design an extended Kalman filter (EKF), referred to as the CEE-EKF, for GPS navigation, providing enhanced robustness against observation outliers.
- The CEE-EKF is proposed as an alternative to the traditional MMSE-based EKF, offering improved robustness and superior estimation performance when handling impulse noise in GPS navigation. The results provide valuable insights into improving the accuracy and reliability of GPS navigation in realistic and challenging noise environments.
- The application of CEE in robust GPS EKF design remains limited in the open literature. The proposed CEE-EKF demonstrates significant improvements in estimation accuracy, and its integration into GPS navigation filter design is detailed in this paper.
- While this study focuses on GPS navigation, the CEE-EKF algorithm can be readily extended to other GNSSs, such as Galileo, BeiDou, and GLONASS, without loss of generality.

The remainder of this paper is organized as follows. A review of the MEE-EKF and MCC-EKF is introduced in Section 2 and Section 3, respectively. In Section 4, the CEE-EKF algorithm is presented. The proposed CEE-EKF’s performance compared to the EKF, MEE-EKF, and MCC-EKF techniques are assessed using illustrative examples based on simulation experiments in Section 5. Finally, conclusions are given in Section 6.

## 2. Minimum Error Entropy Extended Kalman Filter

The minimum error entropy (MEE) criterion is realized by minimizing the second-order Rényi entropy of the estimation error. This criterion is particularly valuable as it facilitates more accurate state estimation, especially in the presence of non-Gaussian measurement errors that frequently occur in practical, real-world scenarios.

In information theory, Rényi entropy is a generalized form encompassing a range of measures. Alfred Rényi introduced this concept in 1961 [5], significantly extending Shannon entropy and Kullback–Leibler (KL) divergence. While Shannon entropy is a specific case of first-order Rényi entropy, commonly used for measuring uncertainty, KL divergence is an asymmetric measure that quantifies the difference between two probability distributions. It is also known as information divergence or relative entropy.

To define the error e between two random variables, X and Y, when α=2, the second-order Rényi entropy is expressed as

(1) $$H_{2}(e)=-\log(\sum_{i}p^{2}(e_{i}))$$

where $p(e_{i})$ represents the probability distribution of the error. This step is fundamental in the context of satellite navigation as it helps to measure the uncertainty in the estimation errors more comprehensively than traditional Gaussian-based methods.

The cost function $J_{MEE}$ is defined as

(2) $$J_{MEE}(x_{k})=\frac{1}{L^{2}}\sum_{i=1}^{L}\sum_{j=1}^{L}G_{\sigma }(e_{j,k}-e_{i,k})$$

This equation calculates the cost associated with the error distribution for the system state $x_{k}$. It is a crucial component in the MEE criterion, which is designed to minimize this cost function by adjusting the state estimate ${\hat{x}}_{k|k}$. Next, the state estimate ${\hat{x}}_{k|k}$ is updated by finding the value that minimizes the cost function:

(3) $${\hat{x}}_{k|k}=\arg\underset{x_{k}}{\max}\;J_{\mathrm{MEE}}(x_{k})=\arg\underset{x_{k}}{\max}\sum_{i=1}^{L}\sum_{j=1}^{L}G_{\sigma }(e_{j,k}-e_{i,k})$$

This optimization process is essential in satellite navigation, where the goal is to refine the state estimates continuously to achieve the most accurate position, velocity, and time (PVT) solution. The gradient of the cost function with respect to the state $x_{k}$ is given by

(4) $$\begin{array}{c} \frac{\partial J_{\mathrm{MEE}}(x_{k})}{\partial x_{k}}=\frac{1}{L^{2}\sigma^{2}}\sum_{i=1}^{L}\sum_{j=1}^{L}\left([e_{j,k}-e_{i,k}]G_{\sigma }(e_{j,k}-e_{i,k})[w_{j,k}-w_{i,k}]\right)\\ =\Gamma_{1}-\Gamma_{2}-\Gamma_{3}+\Gamma_{4}=2\Gamma_{1}-2\Gamma_{3}=0 \end{array}$$

Here, the gradient is used to guide the optimization process, ensuring that the state estimate moves towards the minimum cost. The terms $\Gamma_{1}$ through $\Gamma_{4}$ are defined as

(5) $$\Gamma_{1}=\sum_{i=1}^{L}\sum_{j=1}^{L}e_{j,k}G_{\sigma }(e_{j,k}-e_{i,k})w_{j,k}^{T}$$

(6) $$\Gamma_{2}=\sum_{i=1}^{L}\sum_{j=1}^{L}e_{i,k}G_{\sigma }(e_{j,k}-e_{i,k})w_{j,k}^{T}$$

(7) $$\Gamma_{3}=\sum_{i=1}^{L}\sum_{j=1}^{L}e_{j,k}G_{\sigma }(e_{j,k}-e_{i,k})w_{i,k}^{T}$$

(8) $$\Gamma_{4}=\sum_{i=1}^{L}\sum_{j=1}^{L}e_{i,k}G_{\sigma }(e_{j,k}-e_{i,k})w_{i,k}^{T}$$

These equations represent the weighted sums of the errors, which are key in the derivation of the state update equation. Equations (5)–(8) can be rewritten as

(9) $$\Gamma_{1}-\Gamma_{3}=\frac{1}{L^{2}\sigma^{2}}W_{k}^{T}\Xi_{k}e_{k}-\frac{1}{L^{2}\sigma^{2}}W_{k}^{T}\Psi_{k}e_{k}=0$$

where

(10) $${\left[\Xi_{k}\right]}_{ij}=G_{\sigma }(e_{j,k}-e_{i,k})$$

These matrices play a crucial role in the computation of the cost function, representing the influence of each error term on the overall state estimate.

(11) $$\Psi_{k}=\mathrm{diag}\{\sum_{i=1}^{L}G_{\sigma }(e_{1,k}-e_{i,k}),\ldots ,\sum_{i=1}^{L}G_{\sigma }(e_{L,k}-e_{i,k})\}$$

The matrix $C_{k}$ is given by

(12) $$C_{k}=\Xi_{k}-\Psi_{k}=\left[\begin{array}{cc} C_{x,k} & C_{yx,k}\\ C_{xy,k} & C_{y,k} \end{array}\right]$$

where the submatrices $C_{x,k}$, $C_{xy,k}$, $C_{yx,k}$ and $C_{y,k}$ are defined as

(13) $$\underset{\left(i=1,2,\ldots ,n;j=1,2,\ldots ,n\right)}{C_{x,k}={\left(C_{ij,k}\right)}_{n\times n}={\left(\Xi_{ij,k}\right)}_{n\times n}-{\left(\Psi_{ij,k}\right)}_{n\times n}}$$

(14) $$\underset{\left(i=n+1,n+2,\ldots ,n+m;j=1,2,\ldots ,n\right)}{C_{xy,k}={\left(C_{ij,k}\right)}_{m\times n}={\left(\Xi_{ij,k}\right)}_{m\times n}-{\left(\Psi_{ij,k}\right)}_{m\times n}}$$

(15) $$\underset{\left(i=1,2,\ldots ,n;j=n+1,n+2,\ldots ,n+m\right)}{C_{yx,k}={\left(C_{ij,k}\right)}_{n\times m}={\left(\Xi_{ij,k}\right)}_{n\times m}-{\left(\Psi_{ij,k}\right)}_{n\times m}}$$

(16) $$\underset{\left(i=n+1,n+2,\ldots ,n+m;j=n+1,n+2,\ldots ,n+m\right)}{C_{y,k}={\left(C_{ij,k}\right)}_{m\times m}={\left(\Xi_{ij,k}\right)}_{m\times m}-{\left(\Psi_{ij,k}\right)}_{m\times m}}$$

These submatrices define the relationships between different parts of the state vector, providing a more detailed structure for updating the state estimate. The state update can be rewritten as

(17) $${\hat{x}}_{k}^{t}={\hat{x}}_{k|k-1}+K_{k}^{t-1}[z_{k}-h({\hat{x}}_{k|k-1}^{t-1})]$$

where $K_{k}^{t-1}$ is the gain matrix that determines how much the measurement $z_{k}$ should influence the updated state estimate. This step is analogous to the Kalman gain in traditional filters but adapted for the MEE criterion. The gain matrix $K_{k}^{t-1}$ is calculated as

(18) $$K_{k}^{t-1}={\left(P_{x,k|k-1}^{t-1}+H_{k}^{T}P_{xy,k|k-1}^{t-1}+(P_{yx,k|k-1}^{t-1}+H_{k}^{T}R_{k}^{t-1})H_{k}\right)}^{-1}(P_{yx,k|k-1}^{t-1}+H_{k}^{T}R_{k}^{t-1})$$

This equation integrates the predicted state covariance $P_{x,k|k-1}^{t-1}$, the cross-covariance $P_{xy,k|k-1}^{t-1}$, and the measurement noise covariance $R_{k}^{t-1}$, which, together, determine the optimal weighting of the new measurement in the state update. The individual terms for the covariance matrices are defined as follows:

(19) $$P_{x,k|k-1}^{t-1}={\left(B_{p,k|k-1}^{-1}\right)}^{T}C_{x,k}^{t-1}B_{p,k|k-1}^{-1}$$

(20) $$P_{xy,k|k-1}^{t-1}={\left(B_{r,k}^{-1}\right)}^{T}C_{xy,k}^{t-1}B_{p,k|k-1}^{-1}$$

(21) $$P_{yx,k|k-1}^{t-1}={\left(B_{p,k|k-1}^{-1}\right)}^{T}C_{yx,k}^{t-1}B_{r,k}^{-1}$$

(22) $$R_{k}^{t-1}={\left(B_{r,k}^{-1}\right)}^{T}C_{y,k}^{t-1}B_{r,k}^{-1}$$

Furthermore, the posterior covariance matrix $P_{k}$ can be updated by

(23) $$P_{k}=(I-K_{k}^{t-1}H_{k})P_{k|k-1}^{t-1}{\left(I-K_{k}^{t-1}H_{k}\right)}^{T}+K_{k}^{t-1}R_{k}{\left(K_{k}^{t-1}\right)}^{T}$$

**Figure 1:**
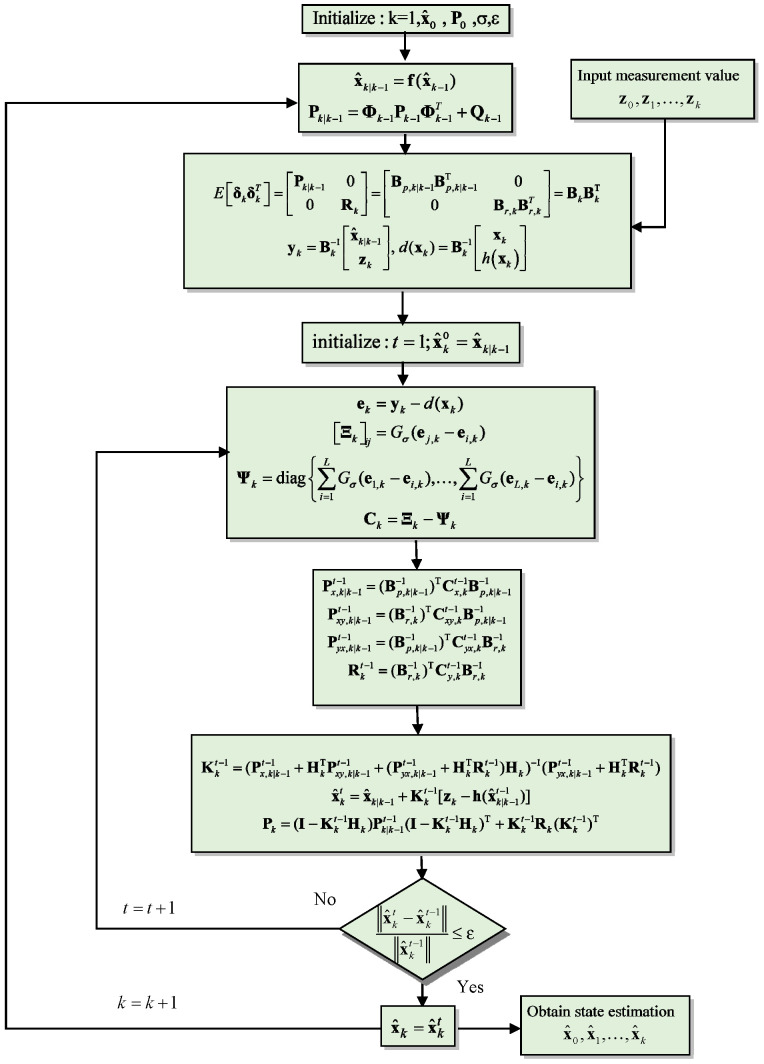
Flowchart of the MEE-EKF algorithm.

In this expression, the matrix $P_{k}$ represents the updated covariance matrix, reflecting the reduced uncertainty in the state estimate after incorporating the measurement $z_{k}$. This update process is critical in satellite navigation applications, where precise and timely updates to the state estimate are essential for accurate positioning and tracking. The flowchart of the MEE-EKF algorithm is shown in Figure 1.

## 3. Maximum Correntropy Criterion Extended Kalman Filter

Correntropy is a measure of similarity between two random variables and has been broadly applied in signal processing, machine learning, and statistics. Unlike the traditional mean square error (MSE) approach, which is sensitive to noise and outliers, correntropy is well-suited for handling non-Gaussian distributions and robustly managing noise and outliers. It achieves this by quantifying similarity through kernel density estimation, offering a more flexible and resilient framework for addressing complex and non-Gaussian noise environments.

The correntropy function can be expressed as

(24) $$V_{\sigma }\left(X,Y\right)=E[\kappa_{\sigma }(X,Y)]=\iint_{x\in X,y\in Y}\kappa_{\sigma }(x,y)f_{XY}(x,y)dxdy$$

where $\kappa_{\sigma }(\cdot )$ is the kernel function that depends on the difference between random variables X and Y. The parameter σ represents the kernel width, while E[·] denotes the expectation value of the kernel function over the joint distribution. The kernel function determines the sensitivity of correntropy to variations between X and Y. The most commonly used kernel function, the Gaussian kernel, is defined as

(25) $$\kappa_{\sigma }(X,Y)=G_{\sigma }(e)=\exp(-\frac{e^{2}}{2\sigma^{2}})$$

$G_{\sigma }$ represents the Gaussian kernel, and e=x−y denotes the difference between the variables X and Y. In practical scenarios, the joint probability distribution $f_{XY}(x,y)$ is usually unknown, so correntropy is estimated from sample averages. The kernel width σ acts as a weighting parameter; however, as it approaches infinity, the MCC converges to the MMSE method. Thus, the estimated correntropy is expressed as

(26) $${\hat{V}}_{\sigma }\left(X,Y\right)=\frac{1}{N}\overset{N}{\underset{i=1}{\sum }}G_{\sigma }(e_{i})=\frac{1}{N}\sum_{i=1}^{N}G_{\sigma }\left(x_{i}-y_{i}\right)$$

The prediction step in the MCC-EKF can be described as

(27) $$\left[\begin{array}{c} {\hat{x}}_{k|k-1}\\ z_{k} \end{array}\right]=\left[\begin{array}{c} x_{k}\\ h(x_{k}) \end{array}\right]+\delta_{k}$$

where ${\hat{x}}_{k|k-1}$ represents the predicted state and $h\left(x_{k}\right)$ is the function that is nonlinear and connects the measurement to the state, and the correction term $\delta_{k}$ is given by

(28) $$\delta_{k}=\left[\begin{array}{c} {\hat{x}}_{k|k-1}-x_{k}\\ v_{k} \end{array}\right]$$

The objective function for the maximum correntropy criterion is defined as

(29) $$J_{\mathrm{MCC}}\left(x_{k}\right)=\frac{1}{L}\sum_{i=1}^{L}G_{\sigma }\left(y_{i,k}-d_{i}(x_{k})\right)$$

This function sums the Gaussian kernel values over L samples, where each kernel value is evaluated at the difference between the error $e_{i,k}$ and the state-dependent divergence $d_{i}(x_{k})$. The goal is to find the state $x_{k}$ that maximizes this objective function, thereby minimizing the divergence between the predicted and actual states.

To find the optimal state estimate, the MCC-EKF solves the following optimization problem:

(30) $${\hat{x}}_{k}=\arg\underset{x_{k}}{\max}\;J_{\mathrm{MCC}}(x_{k})=\arg\underset{x_{k}}{\max}\sum_{i=1}^{L}G_{\sigma }(e_{i,k})$$

The gradient of the objective function with respect to ${\hat{x}}_{k}$ is set to zero to find the maximum:

(31) $$\frac{\partial J_{\mathrm{MCC}}(x_{k})}{\partial x_{k}}=\sum_{i=1}^{L}\left[G_{\sigma }(e_{i,k})w_{i,k}^{T}(y_{i,k}-d_{i}(x_{k}))\right]=0$$

This condition is essential for deriving the update equations in the MCC-EKF. Next, the covariance matrices $C_{k}$ used in the state estimation are structured as follows:

(32) $$C_{k}=\left[\begin{array}{cc} C_{x,k} & 0\\ 0 & C_{y,k} \end{array}\right]$$

The state covariance $C_{x,k}$ and measurement covariance $C_{y,k}$ are defined as

(33) $$C_{x,k}=\mathrm{diag}(G_{\sigma }(e_{1,k}),\ldots ,G_{\sigma }(e_{n,k}))$$

(34) $$C_{y,k}=\mathrm{diag}(G_{\sigma }(e_{n+1,k}),\ldots ,G_{\sigma }(e_{n+m,k}))$$

With the covariance matrices defined, the state update equation for the Kalman filter is expressed as

(35) $${\hat{x}}_{k}^{t}={\hat{x}}_{k|k-1}+K_{k}^{t-1}[z_{k}-h({\hat{x}}_{k|k-1}^{t-1})]$$

The Kalman gain $K_{k}^{t-1}$ is determined by

(36) $$K_{k}^{t-1}=P_{x,k|k-1}^{t-1}H_{k}{\left(H_{k}P_{x,k|k-1}^{t-1}H_{k}^{T}+R_{k}^{t-1}\right)}^{-1}$$

where $P_{k|k-1}^{t-1}$ is the predicted covariance matrix, $H_{k}$ is the measurement matrix, and $R_{k}^{t-1}$ is the measurement noise covariance. These matrices are updated as follows:

(37) $$P_{k|k-1}^{t-1}=B_{p,k}{\left(C_{x,k}^{t-1}\right)}^{-1}B_{p,k}^{T}$$

(38) $$R_{k}^{t-1}=B_{r,k}{\left(C_{y,k}^{t-1}\right)}^{-1}B_{r,k}^{T}$$

In this context, $B_{p,k}$ and $B_{r,k}$ are transformation matrices, with $C_{y,k}^{t-1}$ and $C_{y,k}^{t-1}$ representing the inverses of the state covariance and measurement covariance matrices, respectively. These transformations ensure that the covariance matrices $P_{k|k-1}^{t-1}$ and $R_{k}^{t-1}$ are accurately updated, reflecting the latest state and measurement information.

Finally, the updated state covariance $P_{k}$ is given by

(39) $$P_{k}=(I-K_{k}^{t-1}H_{k})P_{k|k-1}^{t-1}{\left(I-K_{k}^{t-1}H_{k}\right)}^{T}+K_{k}^{t-1}R_{k}{\left(K_{k}^{t-1}\right)}^{T}$$

**Figure 2:**
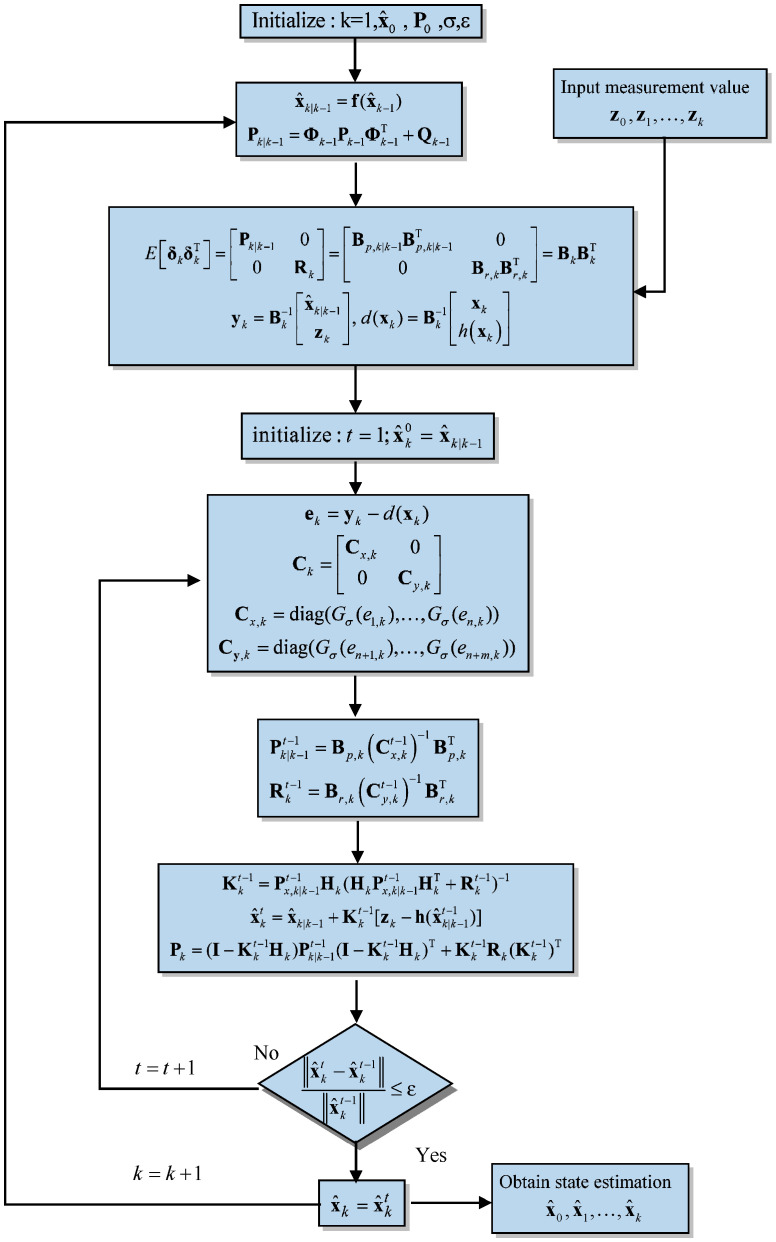
Flowchart of the MCC-EKF algorithm.

This equation provides the final update for the posterior covariance matrix, accounting for the new measurements and their associated uncertainties. This condition is vital for deriving the update equations in the maximum correntropy criterion extended Kalman filter. It ensures that all samples are appropriately balanced in the estimation process, which is crucial for the filter’s robustness and accuracy. By accounting for the nonlinear relationship between the state and measurements, the MCC-EKF can effectively manage non-Gaussian noise and outliers, resulting in improved state estimation. Figure 2 Illustrates the flowchart of the MCC-EKF algorithm.

## 4. Centered Error Entropy Extended Kalman Filter

In filtering techniques, particularly in non-Gaussian noise environments, centered error entropy (CEE) plays a crucial role in addressing the limitations of traditional methods like MEE and MCC. While MEE minimizes error entropy, it often suffers from stability and convergence issues. Conversely, MCC improves error correlation but may not sufficiently reduce uncertainty. CEE merges the strengths of both approaches, offering a more robust and reliable filtering performance by managing higher-order error statistics. Based on their research, Yang et al. [33,34,35,36,37] introduced the CEE-EKF to tackle complex nonlinear problems, thereby enhancing convergence and stability. This approach was later evolved into a weighted version, the MEE-EKF, specifically designed to improve performance in non-Gaussian noise scenarios.

The cost function based on the CEE criterion is defined as follows:

(40) $$J_{\mathrm{CEE}}\left(x_{k}\right)=\lambda \frac{1}{L}\sum_{i=1}^{L}G_{\sigma_{1}}(e_{i})+(1-\lambda )\frac{1}{L^{2}}\sum_{i=1}^{L}\sum_{j=1}^{L}G_{\sigma_{2}}(e_{i}-e_{j})$$

Let λ be a weighting factor used to adjust the ratio between MCC and MEE. The variances $\sigma_{1}$ and $\sigma_{2}$ correspond to MCC and MEE, respectively. The values for $\lambda_{1}$ and $\lambda_{2}$ are defined as

(41) $$\lambda_{1}=\lambda \frac{1}{L}$$

(42) $$\lambda_{2}=\frac{1-\lambda }{L^{2}}$$

These two parameters balance the contributions of the MCC and MEE criteria in the overall cost function. The optimal solution $x_{k}$ can be obtained through maximizing the following objective function, which is the summation of the weighted components of the CEE cost function:

(43) $$\begin{array}{c} {\hat{x}}_{k|k}=\arg\underset{x_{k}}{\max}\;J_{\mathrm{CEE}}(x_{k})\\ \;\;=\arg\underset{x_{k}}{\max}(\lambda_{1}\sum_{i=1}^{L}G_{\sigma }(e_{i,k})+\lambda_{2}\sum_{i=1}^{L}\sum_{j=1}^{L}G_{\sigma }(e_{j,k}-e_{i,k})) \end{array}$$

In this expression, $e_{k}=y_{k}-d(x_{k})$ denotes the difference between the measured value and the predicted value based on the current state $x_{k}$. The optimal solution $x_{k}$ is calculated as follows:

(44) $$\frac{\partial }{\partial x_{k}}J_{\mathrm{CEE}}(x_{k})=\lambda_{1}\sum_{i=1}^{L}\left[G_{\sigma }(e_{i,k})w_{i,k}^{T}(y_{i,k}-d_{i}(x_{k}))\right]+\lambda_{2}\cdot \frac{1}{\sigma_{2}^{2}}\cdot (\Gamma_{1}-\Gamma_{2}-\Gamma_{3}+\Gamma_{4})=0$$

where $w_{j,k}$ and $w_{i,k}$ are weight matrices associated with the state $x_{k}$, and $G_{\sigma }$ represents the gain applied to each term in the cost function. Next, by simplifying the terms in the derivative, we introduce matrices $\Gamma_{1}$, $\Gamma_{2}$, $\Gamma_{3}$ and $\Gamma_{4}$ to represent the intermediate steps:

(45) $$\Gamma_{1}=\sum_{i=1}^{L}\sum_{j=1}^{L}e_{j,k}G_{\sigma_{2}}(e_{j,k}-e_{i,k})w_{j,k}^{T}$$

(46) $$\Gamma_{2}=\sum_{i=1}^{L}\sum_{j=1}^{L}e_{i,k}G_{\sigma_{2}}(e_{j,k}-e_{i,k})w_{j,k}^{T}$$

(47) $$\Gamma_{3}=\sum_{i=1}^{L}\sum_{j=1}^{L}e_{j,k}G_{\sigma_{2}}(e_{j,k}-e_{i,k})w_{i,k}^{T}$$

(48) $$\Gamma_{4}=\sum_{i=1}^{L}\sum_{j=1}^{L}e_{i,k}G_{\sigma_{2}}(e_{j,k}-e_{i,k})w_{i,k}^{T}$$

These matrices simplify the expression for the $J_{\mathrm{CEE}}(x_{k})$ derivative, leading to the final update rule for the state estimate $x_{k}$:

(49) $$\begin{array}{c} \frac{\partial }{\partial x_{k}}J_{\mathrm{CEE}}(x_{k})=\lambda_{1}W_{k}^{T}C_{k}e_{k}+\frac{2\lambda_{2}}{\sigma_{2}^{2}}W_{k}^{T}(\Xi_{k}-\Psi_{k})e_{k}\\ \;\;\;\;\;\;\;=W_{k}^{T}(\lambda_{1}C_{k}+\frac{2\lambda_{2}}{\sigma_{2}^{2}}(\Xi_{k}-\Psi_{k}))e_{k}\\ \;\;\;\;\;\;\;=W_{k}^{T}M_{k}e_{k}\\ \;\;\;\;\;\;\;=0 \end{array}$$

where $C_{k}$, $\Xi_{k}$ and $\Psi_{k}$ are defined as

(50) $$C_{k}=\mathrm{diag}(G_{\sigma_{1}}(e_{1,k}),\ldots ,G_{\sigma_{1}}(e_{L,k}))$$

(51) $${\left[\Xi_{k}\right]}_{ij}=G_{\sigma }(e_{j,k}-e_{i,k})$$

(52) $$\Psi_{k}=\mathrm{diag}\{\sum_{i=1}^{L}G_{\sigma }(e_{1,k}-e_{i,k}),\ldots ,\sum_{i=1}^{L}G_{\sigma }(e_{L,k}-e_{i,k})\}$$

Equation (53) defines the matrix $M_{k}$, which is composed of two main components: the weighting matrix $C_{k}$, whose diagonal elements are based on the gains of the error terms, and the term $\Xi_{k}-\Psi_{k}$. The matrix $M_{k}$ integrates these components to form the basis for updating the state estimation through the gain matrix $K_{k}^{t-1}$:

(53) $$M_{k}=\lambda_{1}C_{k}+\frac{2\lambda_{2}}{\sigma_{2}^{2}}(\Xi_{k}-\Psi_{k})=\left[\begin{array}{cc} M_{x,k} & M_{yx,k}\\ M_{xy,k} & M_{y,k} \end{array}\right]$$

Finally, the state update can be written as

(54) $${\hat{x}}_{k}^{t}={\hat{x}}_{k|k-1}+K_{k}^{t-1}[z_{k}-h({\hat{x}}_{k|k-1}^{t-1})]$$

where the gain matrix $K_{k}^{t-1}$ and the covariance update matrices are defined by

(55) $$K_{k}^{t-1}={\left(P_{x,k|k-1}^{t-1}+H_{k}^{T}P_{xy,k|k-1}^{t-1}+(P_{yx,k|k-1}^{t-1}+H_{k}^{T}R_{k}^{t-1})H_{k}\right)}^{-1}(P_{yx,k|k-1}^{t-1}+H_{k}^{T}R_{k}^{t-1})$$

Equations (56)–(59) detail the specific components of the covariance matrices used in $K_{k}^{t-1}$. These include the inverse state covariance matrix $P_{x,k|k-1}^{t-1}$, calculated using the matrix $B_{p,k|k-1}^{-1}$ and state prediction matrix $M_{x,k}^{t-1}$. The cross-covariance matrix $P_{xy,k|k-1}^{t-1}$ and the measurement noise covariance $R_{k}^{t-1}$ are similarly defined using transformations of these matrices, ensuring that the gain matrix reflects both state and measurement uncertainties.

(56) $$P_{x,k|k-1}^{t-1}={\left(B_{p,k|k-1}^{-1}\right)}^{T}M_{x,k}^{t-1}B_{p,k|k-1}^{-1}$$

(57) $$P_{xy,k|k-1}^{t-1}={\left(B_{r,k}^{-1}\right)}^{T}M_{xy,k}^{t-1}B_{p,k|k-1}^{-1}$$

(58) $$P_{yx,k|k-1}^{t-1}={\left(B_{p,k|k-1}^{-1}\right)}^{T}M_{yx,k}^{t-1}B_{r,k}^{-1}$$

(59) $$R_{k}^{t-1}={\left(B_{r,k}^{-1}\right)}^{T}M_{y,k}^{t-1}B_{r,k}^{-1}$$

This matrix is updated using the gain matrix $K_{k}^{t-1}$ and the observation model, ensuring that, even after taking into account the new observations, the state estimate remains accurate and properly calibrated. This concludes the state update process, followed by the posterior covariance matrix update:

(60) $$P_{k}=(I-K_{k}^{t-1}H_{k})P_{k|k-1}^{t-1}{\left(I-K_{k}^{t-1}H_{k}\right)}^{T}+K_{k}^{t-1}R_{k}{\left(K_{k}^{t-1}\right)}^{T}$$

**Figure 3:**
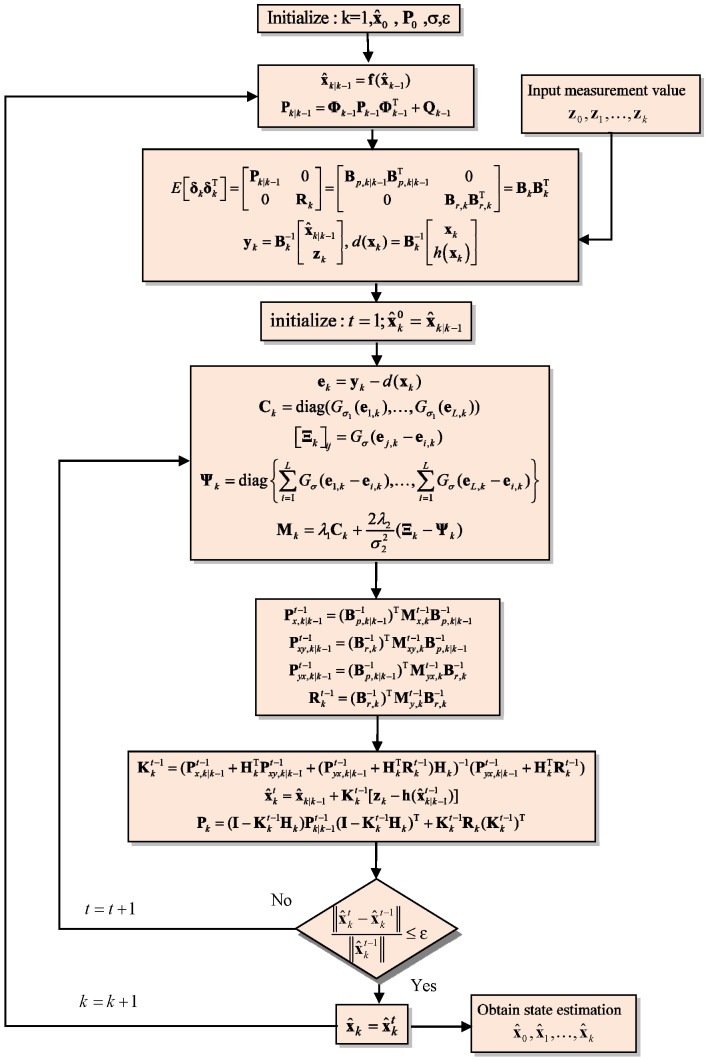
Flowchart of the CEE-EKF algorithm.

Finally, Equation (60) provides the expression for the posterior covariance matrix $P_{k}$, which reflects the most recent and accurate estimate of the system’s state. The flowchart of the CEE-EKF algorithm is shown in Figure 3.

## 5. Results and Discussion

The experiment focuses on the state estimation problem for GPS navigation satellites, where non-Gaussian noise is introduced to assess the filtering performance of several algorithms. The filters tested and compared include EKF, MEE-EKF, MCC-EKF, and CEE-EKF. Each filter’s performance is evaluated based on the error probability density function, allowing for a comprehensive comparison of their effectiveness in various non-Gaussian noise scenarios.

The simulations were conducted on a desktop computer equipped with an Intel Core i5-10400 processor (6 cores, 12 threads, up to 4.3 GHz) and 16 GB of DDR4 RAM, representing a capable mid-range system for productivity, content consumption, and entry-level gaming. Storage was provided by an SSD ranging from 256 GB to 1 TB, and depending on the configuration, either Intel UHD 630 integrated graphics or a dedicated GPU such as the NVIDIA GTX 1650. All simulations were conducted in MATLAB R2021b on Microsoft Windows 10.

**Figure 4:**
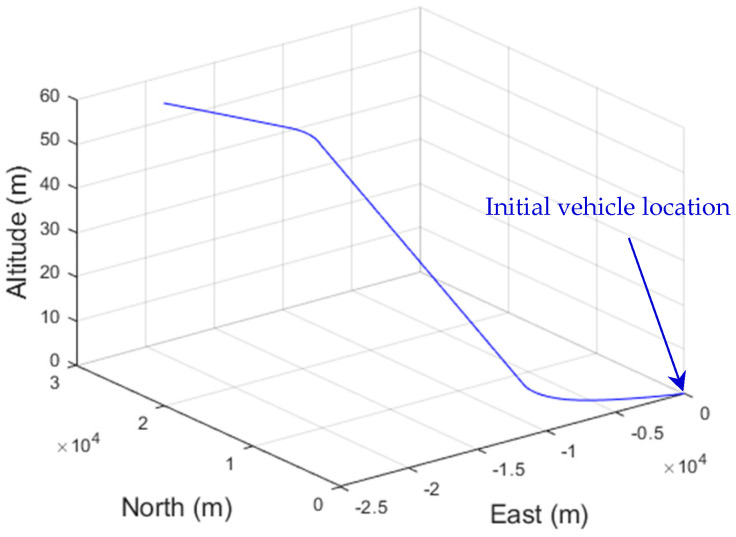
The trajectory plot.

**Figure 5:**
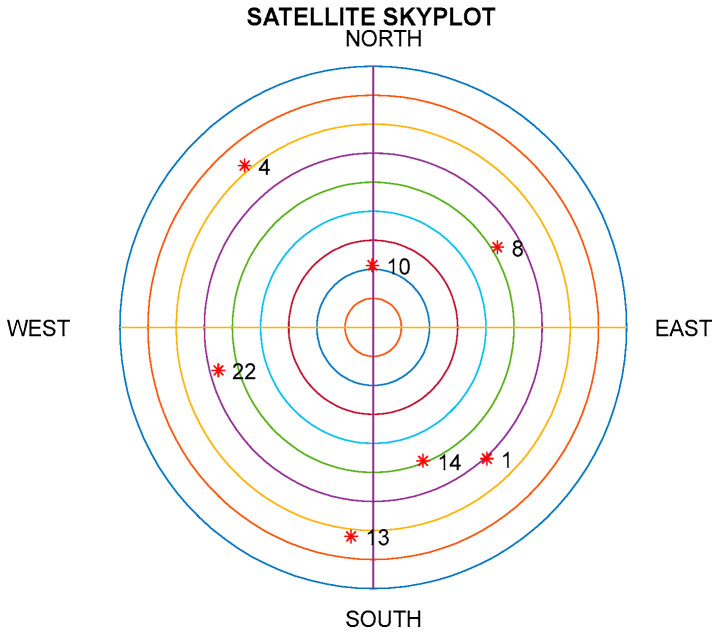
The skyplot for satellites. The red dots (*) indicate the locations of GPS satellites, labeled by their respective GPS ID numbers.

The Satellite Navigation (SatNav) Toolbox 3.0 [43] was employed to generate the data required for GPS positioning, including satellite positions and pseudoranges. The test trajectory and the satellite skyplot for the seven visible GPS satellites (red dots labeled with their GPS ID numbers) of the simulation are shown in Figure 4 and Figure 5, respectively. Positions expressed in geographic coordinates (latitude, longitude, and height) can be converted to WGS-84 Earth-Centered, Earth-Fixed (ECEF) Cartesian coordinates and vice versa. The vehicle taken for simulation is assumed to start from 121.7775 degrees east longitude and 25.1492 degrees north latitude with an initial altitude of 100 m, which is at ${\left[\begin{array}{ccc} -3042329.20 & 4911080.20 & 2694074.30 \end{array}\right]}^{T}$ m at the WGS-84 ECEF coordinate frame.

The initial vehicle position, specified by known geographic coordinates, is defined as the origin (0, 0, 0) of the local tangent East–North–Up (ENU) frame. Once the vehicle trajectory is represented in ENU coordinates, the positions are transformed into the WGS-84 Earth-Centered, Earth-Fixed (ECEF) Cartesian frame. State estimation is then performed based on this trajectory, and errors are evaluated in the east, north, and altitude directions for the different filter algorithms.

Rather than employing a nonlinear model, $\dot{x}=f(x,t)+u$, the dynamics of a GPS receiver in a low-dynamic environment can be described using an eight-state PV (position–velocity) model. The state equations represent the fundamental differential equations of motion, and the process model can be expressed as $\dot{x}=Fx+Gu$. In this case, the GPS navigation filter includes three position components (east, north, and vertical: $x_{1}$, $x_{3}$, and $x_{5}$), three velocity components (east, north, and vertical: $x_{2}$, $x_{4}$, and $x_{6}$), and two clock states ($x_{7}$ and $x_{8}$), resulting in an 8×1 state vector: $x={\left[\begin{array}{cccccccc} x_{1} & x_{2} & x_{3} & x_{4} & x_{5} & x_{6} & x_{7} & x_{8} \end{array}\right]}^{T}$. The state variables in the PV model are driven by white-noise processes: $u={\left[\begin{array}{cccccccc} 0 & u_{2} & 0 & u_{4} & 0 & u_{6} & u_{7} & u_{8} \end{array}\right]}^{T}$.

We define the following submatrices:

$$F_{PV}=F_{clk}=\left[\begin{array}{cc} 0 & 1\\ 0 & 0 \end{array}\right]$$

where the subscripts indicate the submatrices used in the PV and clock models, respectively, and the full F matrix can then be constructed as follows:

$$F=diag(F_{PV},F_{PV},F_{PV},F_{clk})$$

By converting the model to discrete-time form,

(61) $$x_{k+1}=\Phi_{k}x_{k}+w_{k}$$

the state transition matrix can be expressed as

$$\Phi_{k}=diag(\Phi_{k,PV},\Phi_{k,PV},\Phi_{k,PV},\Phi_{k,clk})$$

where the submatrices are identical and given by

$$\Phi_{k,PV}=\Phi_{k,clk}=\left[\begin{array}{cc} 1 & \Delta t\\ 0 & 1 \end{array}\right]$$

where the sampling interval is Δt=1 second. Similarly, for the process noise covariance matrix, the submatrices corresponding to the PV and clock models are defined as

$$Q_{k,PV}=S_{p}\left[\begin{array}{cc} \frac{\Delta t^{3}}{3} & \frac{\Delta t^{2}}{2}\\ \frac{\Delta t^{2}}{2} & \Delta t \end{array}\right];\;Q_{k,clk}=\left[\begin{array}{cc} S_{f}\Delta t+S_{g}\frac{\Delta t^{3}}{3} & S_{g}\frac{\Delta t^{2}}{2}\\ S_{g}\frac{\Delta t^{2}}{2} & S_{g}\Delta t \end{array}\right]$$

and the full process noise covariance matrix can then be constructed as

$$Q_{k}=diag(Q_{k,PV},Q_{k,PV},Q_{k,PV},Q_{k,clk})$$

where $S_{p}$,$S_{f}$, and $S_{g}$ are the respective spectral amplitudes: $S_{p}=50\;{(m/\sec)}^{2}/\left(\mathrm{rad}/\sec\right)$, $S_{f}=0.1\;\sec$ and $S_{g}=0.1\;\sec^{-1}$. These spectral amplitudes can be used to generate the $Q_{k}$ matrix.

By considering the GPS pseudorange observables as the nonlinear measurement of EKF in form of $z_{k}=h(x_{k})+v_{k}$, it can be represented as

(62) $$\rho_{i}=\sqrt{{\left(x_{i}-x\right)}^{2}+{\left(y_{i}-y\right)}^{2}+{\left(z_{i}-z\right)}^{2}}+ct_{b}+v_{\rho_{i}},\;i=1,\ldots ,n$$

where $\left(x_{i},y_{i},z_{i}\right)$ are the three-dimensional i-th satellite’s positions, (x,y,z) is the three dimensions’ user position, the speed of light is c, the receiver clock offset from system time is denoted by $t_{b}$, and $v_{\rho_{i}}$ is the pseudorange noise.

Assuming the pseudorange observables are available, the linearized measurement equation based on n observables for the PV model is given by the following:

(63) $$\underset{z_{k}}{\underbrace{\left[\begin{array}{c} \rho_{1}\\ \rho_{2}\\ \vdots \\ \rho_{n} \end{array}\right]-\left[\begin{array}{c} {\hat{\rho }}_{1}\\ {\hat{\rho }}_{2}\\ \vdots \\ {\hat{\rho }}_{n} \end{array}\right]}}=\underset{H_{k}}{\underbrace{\left[\begin{array}{cccccccc} h_{x}^{\left(1\right)} & 0 & h_{y}^{\left(1\right)} & 0 & h_{z}^{\left(1\right)} & 0 & 1 & 0\\ h_{x}^{\left(2\right)} & 0 & h_{y}^{\left(2\right)} & 0 & h_{z}^{\left(2\right)} & 0 & 1 & 0\\ \vdots & \vdots & \vdots & \vdots & \vdots & \vdots & \vdots & \vdots \\ h_{x}^{\left(n\right)} & 0 & h_{y}^{\left(n\right)} & 0 & h_{z}^{\left(n\right)} & 0 & 1 & 0 \end{array}\right]}}\left[\begin{array}{c} x_{1}\\ x_{2}\\ x_{3}\\ x_{4}\\ x_{5}\\ x_{6}\\ x_{7}\\ x_{8} \end{array}\right]+\left[\begin{array}{c} v_{\rho_{1}}\\ v_{\rho_{2}}\\ \vdots \\ v_{\rho_{n}} \end{array}\right]$$

where $v_{\rho_{1}}$ are additive white-noise measurements. The elements of the measurement model $H_{k}=\partial h(x_{k})/\partial x_{k}$, where $h(x_{k})=\sqrt{{\left(x_{i}-x\right)}^{2}+{\left(y_{i}-y\right)}^{2}+{\left(z_{i}-z\right)}^{2}}+ct_{b}$, involving the direction cosine elements are the partial derivatives of the predicted measurements with respect to each state, which is an (n×8) matrix, respectively.

Outliers in pseudorange observables arise when measurement errors deviate significantly from the predictions of the navigation model. Such outliers typically appear as impulse noise due to transient RF interference or momentary signal obstruction. To evaluate the effectiveness of the CEE-based EKF for GPS navigation, simulations were conducted with injected pseudorange outliers. Two noise patterns were considered: uniformly distributed impulse noise and Gaussian mixture noise. The impulse noise scenario tested the filters’ ability to handle sporadic, high-magnitude disturbances typical of real-world GPS applications, while the Gaussian mixture noise scenario introduced both low- and high-intensity noise to assess robustness across a wider range of conditions. Together, these scenarios enabled a comprehensive comparison of the filters’ noise mitigation capabilities and state estimation accuracy.

To ensure reproducibility of the noise patterns, the random number generator seeds in MATLAB are fixed using randn(‘seed’, 13,579) and rand(‘seed’, 13,579) for generating the Gaussian and uniform random sequences, respectively. This guarantees that the noise injection locations and other random noise patterns are fully reproducible. The initial state and initial covariance are given by $x_{0}={\left[\begin{array}{cccccccc} x_{LS} & 0 & y_{LS} & 0 & z_{LS} & 0 & 0 & 0 \end{array}\right]}^{T}$ and $P_{0}=diag(10^{4},10^{3},10^{4},10^{3},10^{4},10^{3},10^{5},10^{3})$, respectively, where ($x_{LS}$, $y_{LS}$, $z_{LS}$) represents position estimated using the least-squares method.

Monte Carlo simulations were performed based on 100 runs, with performance measured through positioning error and root mean square error (RMSE) to evaluate estimation accuracy. The RMSE formula used in the evaluation is as follows:

(64) $$\mathrm{RMSE}(k)=\sqrt{\frac{1}{N}\sum_{n=1}^{N}{\left(x_{k}-{\hat{x}}_{k}\right)}^{2}}$$

where k represents each step in the single Monte Carlo run; N is the total number of Monte Carlo trials. By calculating the difference between the true value $x_{k}$ and the estimated value ${\hat{x}}_{k}$, the sum of squared errors is computed and square-rooted to obtain the RMSE. This enables a comparison of estimation accuracy among different algorithms.

### 5.1. Scenario 1: GPS Observables with Uniformly Distributed Impulse Noise

Testing of GPS observables with uniformly distributed impulse noise allows us to evaluate the robustness of a filtering algorithm when subjected to extreme and infrequent errors, which are common in real-world GPS signal processing scenarios due to interference or multipath effects. Filters that can effectively suppress these outliers without significant degradation in performance demonstrate higher robustness and reliability, qualities crucial for navigation systems operating in unpredictable scenarios. By introducing impulse noise in this study, we can better assess how well the proposed CEE-EKFs remove these anomalies while maintaining accurate state estimates.

**Figure 6:**
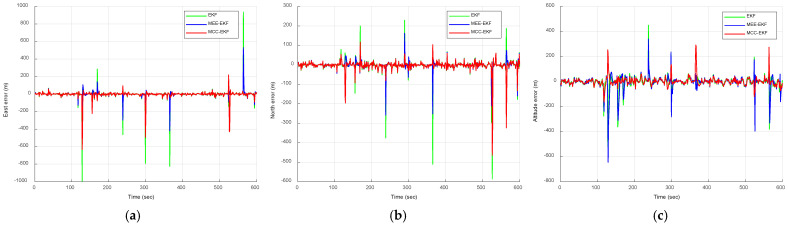
Comparison of positioning errors for EKF, MEE-EKF, and MCC-EKF: (**a**) east; (**b**) north; (**c**) altitude—Scenario 1.

**Figure 7:**
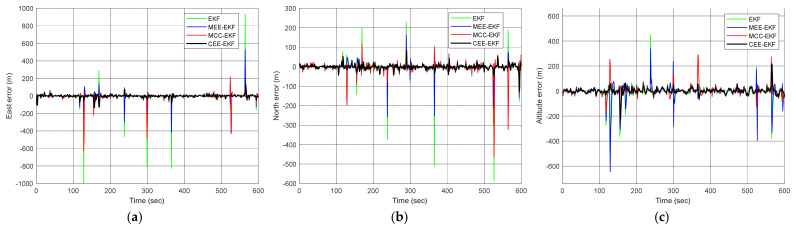
Comparison of positioning errors for the four approaches, EKF, MEE-EKF, MCC-EKF, and CEE-EKF: (**a**) east; (**b**) north; (**c**) altitude—Scenario 1.

The model settings for impulse noise are as follows:

(65) $$r_{k}∼0.98N(0,10)+0.02U(80,200)$$

where N(0,10) and U(80,200) represent a zero-mean Gaussian sequence with a variance of 10 m^2^ and uniformly distributed sequence ranging from 80 to 200 m, occurring with probabilities of 0.98 and 0.02, respectively. The uniformly distributed sequence is regarded as the impulse noise. This model incorporates a 2% proportion of impulse noise to evaluate the outlier resistance capabilities of each filtering algorithm. Figure 6 compares the positioning errors of the EKF, MEE-EKF, and MCC-EKF, while Figure 7 further compares these three filtering algorithms with the CEE-EKF in an impulse noise environment. In the simulation, kernel bandwidths were set for the MEE-EKF, namely $\sigma_{MEE}=4000$ and MCC-EKF: $\sigma_{MCC}=400$, while the CEE-EKF employed a weighting factor λ=0.9 along with two kernel bandwidths: $\sigma_{MEE}=4$ and $\sigma_{MCC}=40$. The GPS test results clearly indicate that the CEE-EKF outperforms the other filters in the east, north, and altitude directions. It consistently exhibits smaller positioning errors and effectively suppresses outliers at critical points, demonstrating superior performance in this noisy environment.

**Figure 8:**
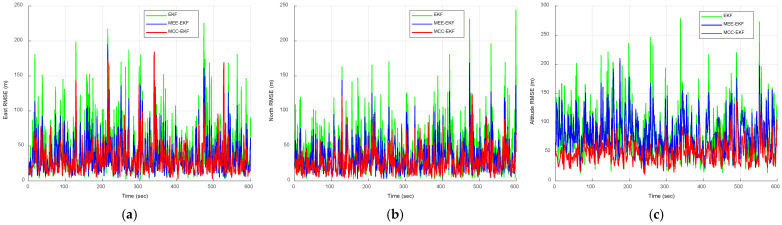
RMSE comparison of EKF, MEE-EKF, and MCC-EKF: (**a**) east; (**b**) north; (**c**) altitude—Scenario 1.

**Figure 9:**
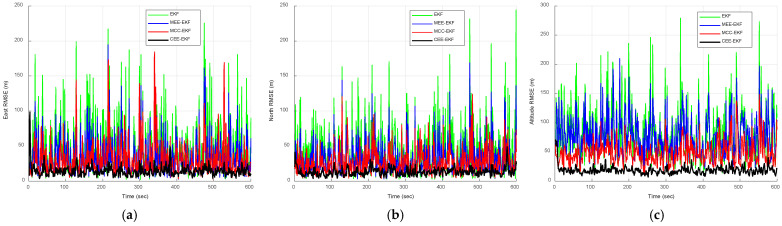
RMSE comparison of for the four approaches, EKF, MEE-EKF, MCC-EKF and CEE-EKF: (**a**) east; (**b**) north; (**c**) altitude—Scenario 1.

The RMSE results obtained from the Monte Carlo simulations are presented in Figure 8 and Figure 9. Figure 8 compares the RMSE performance of the EKF, MEE-EKF, and MCC-EKF, whereas Figure 9 presents the RMSE comparison among all four algorithms. The CEE-EKF achieves significantly lower RMSE values in all directions (east, north, and altitude). The filter rapidly converges to a lower RMSE and maintains superior performance throughout the test. These results confirm that the CEE-EKF is the most effective filter among the four in this environment.

As shown in Table 1, CEE-EKF outperforms the other filters across all directions, demonstrating superior robustness and noise suppression in impulse noise environments. In contrast, EKF struggles the most, particularly with altitude errors. MEE-EKF shows some improvements but still underperforms in certain areas, while MCC-EKF performs better in specific directions but lacks consistency. CEE-EKF is the most effective filter, providing more robust performance under these conditions.

### 5.2. Scenario 2: GPS Observables with Gaussian Mixture Noise Sequence

Gaussian mixture noise, i.e., noise modeled as a mixture of multiple Gaussian distributions, was specifically designed in this experiment because it better represents real-world noise scenarios, where signal disturbances often combine minor, frequent noise and more significant, rarer disturbances. By introducing this Gaussian mixture noise model, we can more accurately simulate these real-world scenarios and evaluate how well the filtering algorithms maintain accuracy under both typical and extreme noise conditions. This setup ensures that the proposed filters are robust enough to handle varying noise intensities and distributions. The Gaussian mixture sequence model is defined as follows:

(66) $$r_{k}∼0.9N(0,100)+0.1N{(0,10}^{7})$$

where N(0,100) and $N{(0,10}^{7})$ represent zero-mean Gaussian sequences with variances of 100 m^2^ and $10^{7}$ m^2^, occurring with probabilities of 0.9 and 0.1, respectively. The Gaussian-distributed sequence with the larger variance is regarded as impulse noise. This model incorporates a 10% proportion of random impulse noise to evaluate the outlier resistance capabilities of each filtering algorithm.

**Figure 10:**
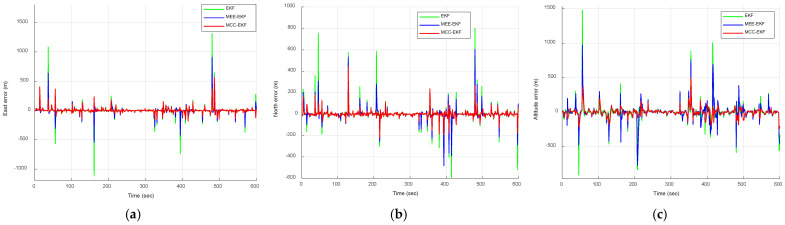
Comparison of positioning errors for EKF, MEE-EKF, and MCC-EKF: (**a**) east; (**b**) north; (**c**) altitude—Scenario 2.

**Figure 11:**
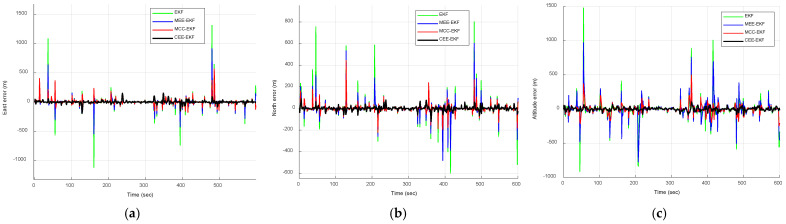
Comparison of positioning errors for the four approaches, EKF, MEE-EKF, MCC-EKF, and CEE-EKF: (**a**) east; (**b**) north; (**c**) altitude—Scenario 2.

Figure 10 compares the positioning errors of the EKF, MEE-EKF, and MCC-EKF, while Figure 11 presents the comparison of these three filtering algorithms with the CEE-EKF in a Gaussian mixture noise environment. As shown in Figure 11, the Gaussian mixture noise highlights clear improvement in positioning errors. The CEE-EKF demonstrates superior robustness and accuracy across all directions, consistently maintaining lower errors even under mixed noise conditions. In contrast, the other filters, particularly the EKF, exhibit larger fluctuations and higher positioning errors, with the differences being more evident in challenging scenarios such as altitude estimation.

**Figure 12:**
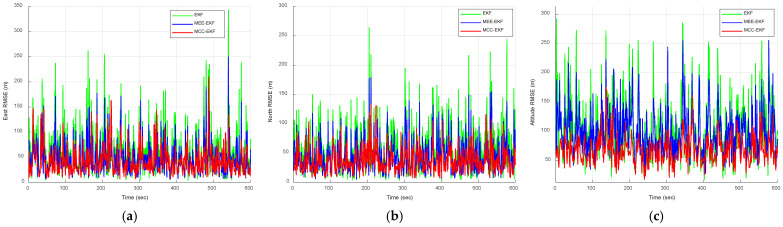
RMSE comparison of EKF, MEE-EKF, and MCC-EKF: (**a**) east; (**b**) north; (**c**) altitude—Scenario 2.

**Figure 13:**
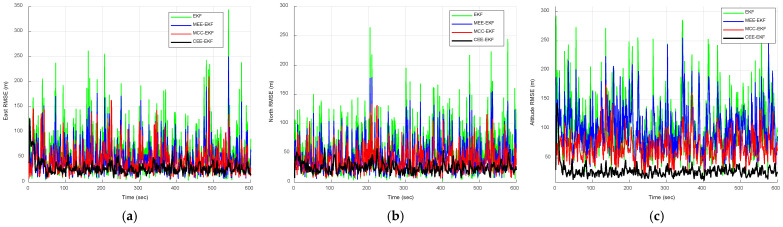
RMSE comparison for the four approaches, EKF, MEE-EKF, MCC-EKF and CEE-EKF: (**a**) east; (**b**) north; (**c**) altitude—Scenario 2.

Similarly, the RMSE results obtained from Monte Carlo simulations are presented in Figure 12 and Figure 13. Figure 12 compares the RMSE performance of the EKF, MEE-EKF, and MCC-EKF, while Figure 13 presents the RMSE comparison among all four algorithms. As shown in Figure 13, the CEE-EKF consistently demonstrates improved performance, exhibiting lower error accumulation over time. Although the other filters are effective in certain aspects, they show less consistency and higher overall RMSE values across different directions. These results indicate that the CEE-EKF provides more reliable noise suppression and better adaptability to varying noise conditions, thereby achieving superior filtering performance. Table 2 summarizes the RMSE values, further confirming that the CEE-EKF remarkably enhances performance in Gaussian mixture noise environments. It consistently maintains both accuracy and robustness throughout the test, highlighting its effectiveness in managing complex noise patterns.

## 6. Conclusions

This study addressed the state estimation problem of GPS navigation under non-Gaussian noise conditions by using the centered error entropy-based extended Kalman filter. Two example scenarios of GPS navigation state estimation with observable outliers were presented, one with uniformly distributed impulse noise and another with Gaussian mixture noise, in order to evaluate whether the proposed filter can more effectively mitigate the effects of non-Gaussian noise. The filtering performance of the EKF, MEE-EKF, MCC, and CEE-EKF was compared in complex, nonlinear noise environments.

The results demonstrate that CEE-EKF consistently outperformed the other algorithms in terms of both noise suppression and estimation accuracy, particularly in GPS environments. While the performance of MEE-EKF was closer to that of EKF, CEE-EKF showed significantly better error reduction and robustness in the presence of non-Gaussian noise. Moreover, CEE-EKF addressed the limitations of MCC and MEE-EKF by enhancing noise suppression and maintaining stable performance, even in challenging GPS conditions. The evaluation using error probability density functions and root mean square error metrics confirmed that CEE-EKF had superior filtering performance. Its ability to effectively handle outliers and quickly adapt to high-intensity noise highlights its practical utility in GPS navigation systems. The findings suggest that CEE-EKF provides enhanced noise suppression and state estimation accuracy in non-Gaussian noise environments. By combining the strengths of both MCC and MEE-EKF, CEE-EKF achieved better error convergence and robustness, making it a reliable and efficient solution for state estimation in complex and noisy environments. This algorithm shows strong potential for future applications in scenarios requiring high-precision positioning under adverse conditions.

Although this paper focuses on GPS navigation, the proposed algorithm can be readily extended to other GNSSs, such as Galileo, BeiDou, and GLONASS, without loss of generality.

Several issues remain important directions for future research:

1. Adaptive determination of free parameters, including the kernel bandwidths ($\sigma_{MEE}$ and $\sigma_{MCC}$) and the weighting factor (λ), for which data-driven or self-tuning strategies would be preferable;
2. Performance comparison with kernels other than the Gaussian kernel, such as Student’s t, Laplace, and Cauchy kernels, as well as combinations thereof;
3. Evaluation under other non-Gaussian pseudorange error models to better characterize NLOS signal reception and multipath interference in real-world environments;
4. Theoretical analysis of the stability and robustness of the proposed method, together with a sensitivity analysis.

## Figures and Tables

**Table 1:** RMSE comparison of different algorithms under a uniformly distributed impulse noise environment.

Filter	RMSE		
	East (m)	North (m)	Altitude (m)
EKF	44.7255	41.7277	81.4417
MEE-EKF ($\sigma_{MEE}=4000$)	34.6927	32.2780	77.7478
MCC-EKF ($\sigma_{MCC}=400$)	32.3500	27.5274	48.6833
CEE-EKF ($\lambda =0.9,\;\sigma_{MEE}=4,\;\sigma_{MCC}=40$)	14.4797	13.1921	18.7295

**Table 2:** Comparison of RMSE for various algorithms under Gaussian mixture noise.

Filter	RMSE		
	East (m)	North (m)	Altitude (m)
EKF	57.6872	54.1626	102.9308
MEE-EKF ($\sigma_{MEE}=4000$)	46.7501	44.1522	97.6033
MCC-EKF ($\sigma_{MCC}=400$)	43.3192	37.5069	67.0332
CEE-EKF (λ=0.9, $\sigma_{MEE}=4$, $\sigma_{MCC}=40$)	28.1809	25.1601	27.7064

## Data Availability

The data that support the findings of this study are available upon reasonable request from the authors.
